# Cognitive–Emotional Aspects of Post-Traumatic Stress Disorder in the Context of Female Genital Mutilation

**DOI:** 10.3390/ijerph19094993

**Published:** 2022-04-20

**Authors:** Nele Wulfes, Uwe von Fritschen, Cornelia Strunz, Nadine Kröhl, Roland Scherer, Christoph Kröger

**Affiliations:** 1 Department of Clinical Psychology and Psychotherapy, University of Hildesheim, 31141 Hildesheim, Germany; nadine.kroehl@tu-braunschweig.de (N.K.); christoph.kroeger@uni-hildesheim.de (C.K.); 2 Helios Klinikum Emil von Behring, 14165 Berlin, Germany; uwe.von-fritschen@helios-gesundheit.de; 3 Desert Flower Center Waldfriede, 14163 Berlin, Germany; c.strunz@waldfriede.de (C.S.); r.scherer@waldfriede.de (R.S.)

**Keywords:** female genital mutilation/cutting, FGM, FGC, post-traumatic stress disorder, age at FGM/C, centrality of event, guilt

## Abstract

Around 200 million women and girls worldwide are affected by female genital mutilation/cutting (FGM/C). FGM/C is a procedure that harms or alters the external female genitals for non-medical reasons, and is usually performed on children. Often, this procedure leads to severe consequences for the women’s physical and mental health. In a cross-sectional sample of 112 women seeking medical counseling, physical and mental health characteristics associated with FGM/C were examined and possible predictors for the development of post-traumatic stress disorder (PTSD) in women affected by FGM/C were identified. A total of 55.4% (*n* = 66) of the women reported symptom levels of probable PTSD. Predictors for higher PTSD symptomology were an older age at the time of the FGM/C procedure, feelings of guilt and the centrality of the event in the woman’s life. Thus, cognitive–emotional processing was found to play an important role in the emergence of PTSD in women suffering from FGM/C. However, interventions taking into consideration these characteristics are mostly lacking and need to be investigated further in the context of FGM/C.

## 1. Introduction

Female genital mutilation/cutting (FGM/C) is a procedure that harms or alters the external female genitals for non-medical reasons. It is usually performed during childhood and often under unsanitary conditions [1]. Consequently, FGM/C has been recognized as an extreme violation of the rights and health of women and girls. In 2015, the international community adopted new development goals that envisage the elimination of FGM/C by 2030 [2]. The World Health Organization (WHO) [1] differentiates four types of FGM/C: type I—partial and/or full removal of the clitoris glans and/or clitoral hood (clitoridectomy); type II—removal of the labia minora with or without partial or full removal of the clitoris glans, with or without excision of the labia majora (excision); type III—narrowing of the vaginal orifice with creation of a covering seal with or without removal of the clitoris glans (infibulation); type IV—all other harmful procedures to the female genitalia for non-medical purposes.

Around 200 million women and girls worldwide are affected by FGM/C. The prevalence rates vary highly depending on the country of origin and even the region within a country [1,3,4]. According to statistics by Terre des Femmes [4], based on the prevalence rates of the countries of origin, the current number of adult women affected in Germany is estimated to be around 74,900. Another 20,200 underage girls in Germany are estimated to be at risk of FGM/C.

FGM/C can lead to severe long-term problems in the urogenital and/or rectovaginal tract. These include fistulas, chronic pain, excessive scarring, an increased frequency of infections (e.g., pelvic infections, urinary tract infections and infections with sexually transmitted diseases), as well as the formation of cysts or neuromas [5,6]. It is assumed that there are risks for all FGM/C types, but that there are differences in severity and prevalence depending on the extent of mutilation [1,7]. Especially with FGM/C type III, prolonged and painful menstruation due to congestion of blood in the vagina (hematocolpos) and uterus (hematometra) or urinary retention as a result of extensive narrowing of the vaginal entrance are frequently seen [1,8]. Furthermore, obstetric complications with increased risk of laceration of the anal sphincter, as well as higher rates of episiotomy, caesarian section and resuscitation of the neonate, are reported, especially when conducted in countries with a lower standard of care [9]. Prolonged labor can lead to the formation of vesico- or rectovaginal defects, resulting in urinary and fecal incontinence [8,10,11].

However, effects on sexuality have been discussed controversially: while sexual functioning does not inevitably need to be impaired, symptoms such as discomfort, pain, burning and bleeding during intercourse, internal and external dyspareunia and decreased sexual satisfaction, as well as rare or absent orgasms, have been reported [6,7,8,12,13,14]. Nevertheless, high-quality research regarding the sexual consequences of FGM/C is still lacking [7,15]: sexual pleasure and satisfaction are multidimensional and causal relationships still need to be examined further [7,14,15].

FGM/C meets criterion A of the Diagnostic and Statistical Manual of Mental Disorders (*DSM-5* [16]) for post-traumatic stress disorder (PTSD; confrontation with actual or threat of death, severe injury or sexual violence). Accordingly, affected women may develop mental disorders, such as affective disorders, anxiety disorders, somatization disorder and PTSD [17,18]. Previous research found that more severe forms of FGM/C are associated with higher levels of mental health symptoms and higher long-term neuro-endocrinological alterations [17,19,20,21,22]. Additionally, Pechmann et al. [20] found a moderating influence of personal attitudes towards FGM/C on the relationship between FGM/C type and mental health symptoms: It was shown that an FGM/C-affirmative attitude was associated with less mental health symptoms. Thus, cognitive–emotional processing might also play an important role in the development of PTSD after FGM/C. Knipscheer et al. [22] showed that an infibulation (type III in accordance with the WHO [1]), a vivid memory of the event and a substance-misusing coping style were associated with enhanced levels of PTSD symptoms. Furthermore, Vloeberghs et al. [19] found that women who had been educated concerning the mutilation, women with whom the procedure was discussed before it happened and women who were older at the time that the FGM/C procedure was performed showed higher PTSD scores. The findings on age at the FGM/C procedure are, however, inconsistent with findings by Köbach et al. [21], who reported higher hair cortisol concentrations for women who were under one year of age when the procedure happened (only FGM/C type I was included). To conclude, research on the underlying factors of the development of PTSD associated with FGM/C is still very limited.

With the development of *DSM-5* [16], the primacy focus on fear and anxiety associated with PTSD has been broadened, taking into consideration further negative emotions and cognitive processing (e.g., anger, guilt and shame). In non-FGM/C populations, shame has been found to be an important predictor for PTSD symptoms [23,24]. Feelings of shame could also be observed in women affected by FGM/C [25] and could play an important role in the development of PTSD-related symptoms. Even though shame and guilt are highly linked and their individual effects are difficult to differentiate [23], some research suggests feelings of guilt as a mediator in the development of psychological distress after traumatic events [26]. Additionally, guilt-related cognitions were associated with PTSD [27]. Usually, FGM/C is done without the consent of the girl or woman. Nevertheless, post-hoc ruminating in a search for control of the situation can lead to cognitive misinterpretations of guilt and self-blame [28]. Moreover, the centrality of a traumatic event in the identity of a person is another important predictor for PTSD symptoms in non-FGM/C populations [23]. The increased centrality of a traumatic event can lead to more intrusive, accessible and vivid memories, and thus a greater post-traumatic burden [23,29]. Additionally, memories that are more central to one’s life story are closely linked to the way people see themselves and others [23]. If FGM/C is more central to a woman’s life story, it could therefore increase the probability of PTSD symptoms. In burn victims, a subjectively more negative body image is assumed to be associated with greater psychological stress—regardless of the actual severity of the injury [30]. FGM/C can also be considered a permanent injury of the body [1]. Additionally, women with FGM/C living in western countries show more mental health symptoms than those still living in their home countries, suggesting an influence of cultural norms on body image [11]. It could therefore be assumed that a more negative attitude towards one’s own body concerning FGM/C is associated with more PTSD symptoms in women suffering from FGM/C.

The aim of the present study was to examine physical and mental health characteristics and factors associated with PTSD symptoms in women who took part in a medical consultation informing them about reconstructive surgery after FGM/C. It was expected that the type of mutilation and the age at which FGM/C was preformed would be associated with an increase in PTSD symptoms. Additionally, cognitive–emotional aspects such as feelings of shame and guilt, the subjective centrality of the event in the life story and thus in the identity of the woman, as well as the attitude towards one’s own body, were expected to be associated with PTSD symptoms in women suffering from FGM/C.

## 2. Materials and Methods

### 2.1. Recruitment and Process

The study was conducted between August 2018 and January 2021. Parts of the sample and the reasons for reconstructive surgery have already been reported by Wulfes et al. [31]. The women were advised and examined in the Desert Flower Center Waldfriede (DFC) as part of a medical consultation. Before the consultation, the participating women were informed about the study, and signed a written agreement for participation. Self-assessment questionnaires were sent to the women by email prior to the consultation. In order to reach as many women as possible, English questionnaires were used. Usually, the women brought the completed questionnaires to the consultation. If the women spoke neither German, Somali nor English, the women’s friends, relatives or social workers helped during translation. Somali-speaking women (most of the participants) had the opportunity to fill out the questionnaires directly before the examination with a language mediator (a Somali nursing assistant with many years of experience working with FGM/C survivors). Additionally, a medical examination was carried out by a surgeon who had coordinated the FGM/C center for many years. When necessary, medical follow-up care, surgical interventions and a self-help group were provided.

Participation in the study was voluntary and had no influence on further medical advice or treatment. The inclusion criterion for the study was the presence of FGM/C as diagnosed by the physician. Exclusion criteria consisted of language barriers when completing the questionnaires or the lack of opportunities for translation.

### 2.2. Sample Characteristics

Of the 193 women who sought medical counseling, 112 (58.0%) consented to participate in the study, and met the inclusion criteria. The mean age was 28.1 years (*SD* = 9.4; 14–63 years), and 23.2% (*n* = 26) of the participants were currently in an intimate relationship. Most of the women came from Somalia (64.3%; *n* = 72), Nigeria (6.3%; *n* = 7) or one of 17 other countries. The mean length of stay in Germany was 38.8 months (*SD* = 50.7; 1–316 months). Detailed socio-demographic sample characteristics can be found in Table 1 and Table S1.

### 2.3. Measures

#### 2.3.1. Depression

The *Patient Health Questionnaire-2* (PHQ-2; [32]), a two-item short form of the depression module of the *Patient Health Questionnaire* [33], was used to screen for depression symptoms. A cut-off value of 3 was used for suspected depressive episodes [32].

#### 2.3.2. Anxiety Disorder

The *Generalized Anxiety Disorder Scale-2* (GAD-2; [34]), a two-item short form of the *Generalized Anxiety Disorder Scale-7* (GAD-7; [34]), was used to screen for anxiety symptoms. In a meta-analysis, a cut-off value of 3 was confirmed reliable for a suspicion of anxiety disorder [35].

#### 2.3.3. Post-Traumatic Stress Disorder

To measure the effects of FGM/C as a traumatic event, the *Primary Care PTSD Screen for DSM-5* (PC-PTSD-5; [36]) was used, which is based on the diagnostic criteria of the *DSM-5* [16]. A cut-off of 3 was established for suspicion of PTSD [36].

#### 2.3.4. Feelings of Shame and Guilt about FGM/C

The subscales *Shame* and *Guilt* of the *State Shame and Guilt Scale* (SSGS; [37]) were used to measure trauma-associated shame and guilt.

#### 2.3.5. Subjective Centrality of FGM/C in Life

The *Centrality of Events Scale—Short Form* (CES; [29]) is a seven-item short form of the *Centrality of Events Scale* [29] and measures how centrally a traumatic event is anchored in the life story and thus the identity of a person.

#### 2.3.6. Body Image or Attitudes to One’s Own Body Regarding FGM/C

In order to record the (positive) body image or the (positive) attitude towards one’s own body before and after surgery, based on Pusic et al. [38], the *Body Image Scale* was formed using nine items.

#### 2.3.7. Reliability of the Measures

In the present study, Cronbach’s *α* values were acceptable to high (PHQ-2 = 0.76; GAD-2 = 0.81; PC-PTSD-5 = 0.78; SSGS total test = 0.88; SSGS subscale *Shame* = 0.78; SSGS subscale *Guilt* = 0.87; CES = 0.74; *Body Image Scale* = 0.82).

### 2.4. Data Analysis

The data were analyzed using IBM SPSS Statistics (Version 26, IBM, Armonk, NY, USA) for Windows. The women were included in the study if both the medical diagnosis and a maximum of 20% missing values in the relevant questionnaires were present. Little’s MCAR test (Missing Completely At Random [39]) was non-significant, χ^2^ _(1147; N = 112)_ = 1091.10 *p* = 0.880, i.e., missing values occurred completely at random. Accordingly, the missing values in the relevant questionnaires were estimated using the maximum likelihood method (expectation-maximization (EM) algorithm). Unless otherwise stated, a probability of a Type I error of 5% (α = 0.05) was assumed. To compare differences between women who met the criteria for suspected symptoms of PTSD and women who did not, and among the types of FGM/C, χ^2^- and *t*-tests were calculated based on all valid cases. If the expected frequencies were below 5, the exact Fischer test was used instead of the χ^2^- test. After the Bonferroni correction, socio-demographic differences were considered significant if *p* < 0.003 and health consequences if *p* < 0.004. Effect sizes were determined using Cohen’s *d* and Cramer’s *V*. Predictors for the suspected diagnosis of PTSD were analyzed using hierarchical multiple regression analysis. In the first step, the characteristics “circumcision type” (dummy-coded) and “age at FGM/C” were added. In the second step, the characteristics “shame”, “guilt” and the “subjective centrality of FGM/C in life” followed. In the third step, the predictor “attitude towards one’s own body in relation to FGM/C” was included in the analysis. We also checked the requirements for regression analysis by reviewing the scatterplots to check the assumptions of linearity and homoscedasticity, as well as to screen for outliers and obvious unusual cases. Variance inflation factors (VIF) were calculated to rule out possible multicollinearity of the predictors. The Durbin–Watson test was used to examine the assumption of independent errors.

## 3. Results

### 3.1. Medical Characteristics

FGM/C took place at a mean age of 7.3 years (*SD* = 3.5; range: 0–21 years). According to the medical examination, 18.8% (*n* = 21) of women were diagnosed with type I FGM/C, 31.3% (*n* = 35) with type II and 50.0% (*n* = 56) with type III. Of 112 women, 59.9% (*n* = 67) reported having pain in the lower stomach “always” or “often.” A total of 56.2% (*n* = 63) reported menstrual pain, 25.0% (*n* = 28) reported straining to empty the bladder, 22.3% (*n* = 25) had pain urinating, 18.7% (*n* = 11) had gynecological infections, 17.8% (*n* = 20) reported stagnation of blood during periods, and 16.1% (*n* = 18) experienced urinary infections. Overall, menstruation was the body function that caused the severest health complaints or impairments (62.5%; *n* = 70). Generally, “quite a lot” or “very much” in terms of impairment in daily life and social withdrawal was reported by 60.8% (*n* = 68) and 37.9% (*n* = 43) of women. Detailed results on medical complications can be found in Table 2 and Table S2. After Bonferroni correction, no differences in physical health consequences between the different types of FGM/C were found.

### 3.2. Mental Health Characteristics

Based on the self-report measures, a depressive episode was suspected in 36.6% (*n* = 41), anxiety disorder in 48.2% (*n* = 54) and PTSD in 55.4% (*n* = 62; see Table 1). Of the participants with suspected PTSD, 45.2% (*n* = 28) participants were suspected to fulfill the criteria of a comorbid depressive episode, and 64.5% (*n* = 40) to fulfill the criteria of a comorbid anxiety disorder. After Bonferroni correction (*p* < 0.003), there was only a significant difference in terms of suspected anxiety disorder (*p* ≤ 0.001; Cramer’s *V* = 0.36) between women with and without suspected PTSD.

### 3.3. Predictors of Suspected PTSD

The results of the hierarchical multiple regression analysis are shown in Table 3. A correlation matrix of all analysis-relevant characteristics can be found in Table S3. In the first step of the analysis, only higher age at the FGM/C procedure was associated with a higher level of PTSD symptoms, explaining 6% of the variance (R^2^ = 0.06; F_(3, 108)_ = 2.10; *p* = 0.105). In the second step, “shame,” “guilt” and “centrality of the event in life” were included. These additionally explained 30% of the variance, and led to a significant change (∆R^2^ = 0.30, ∆F_(3, 105)_ = 16.45; *p* < 0.001). Adding the “attitude towards one’s own body” in a third step did not lead to any significant change (∆R^2^ = 0.01, ∆F_(1, 104)_ = 2.19; *p* = 0.142). Overall, the model explained 33% of the variance in the PTSD symptoms. A closer look at the individual predictors in the final model showed that the age at which FGM/C was performed (*p* = 0.045) and the level of “guilt” (*p* = 0.006) and of “centrality of the event in life” (*p* = 0.035) were significant predictors for PTSD symptoms.

## 4. Discussion

The aim of the present study was to determine predictors for PTSD symptoms in women suffering from FGM/C. Compared to previous research, the rate of suspected PTSD in this study was quite high (55.4% vs. 16–44.3%; [18]). In the present study, suspected PTSD was assessed using screening instruments, as is done by most studies assessing PTSD in women suffering from FGM [17]. Nonetheless, using screening instruments rather than full diagnostic criteria can lead to higher rates of suspected PTSD. Since the study took place in the context of a medical examination in a specialized department, it is possible that women who were more impaired by FGM/C were more likely to participate. In the present study, 50.0% of the women were found to have undergone FGM/C type III. This rather high proportion roughly corresponds to the prevalence rates in other studies (46.8%; [20] and 54%; [19,22]). Additionally, type III is mainly practiced in the northeastern regions of Africa [19]. Since most women (64.3%) in the current sample came from Somalia, another reason for the high prevalence can be the country of origin. In accordance with previous research [5,6,7], several medical complications—such as pain in the abdomen (59.9%), menstrual pain (56.2%) and pain to empty the bladder (25.0%), etc.—were reported. However, no significant differences between the types of FGM/C could be found.

Regression analysis showed the age at which FGM/C was performed, feelings of guilt and the centrality of the event in life to be significant predictors for the severity of the PTSD symptoms. The final model in the present study explained 33% of the variance. Contrary to previous findings [17,19,20,21,22], the type of FGM/C was not a significant predictor for the development of PTSD symptoms. In the present study, a higher age at the FGM/C procedure was associated with a higher level of PTSD symptoms, and this adds information to the inconsistent findings [19,21]. It could be argued that women on whom FGM/C is performed at an older age are more likely to remember the procedure, and may perceive the intervention as a violation of self-determination and betrayal by caregivers and family members.

The disseminated attitudes and norms of the socio-cultural context (i.e., peers and relatives) may have an important impact on the symptom level. For example, women who have undergone FGM/C and are currently still living in their home countries show fewer mental health symptoms than those living in western culture [11]. Markedly, a previous study revealed that a positive attitude towards FGM was associated with fewer symptoms of depression and PTSD [20]. Nonetheless, contrary to our expectations, a more negative attitude towards one’s own body concerning FGM/C was not a significant predictor for PTSD symptoms.

Similar to findings in populations who experienced other traumatic events [23,27,29], indicators of dysfunctional cognitive–emotional processing, such as feelings of guilt and the centrality of the event in life, were associated with higher PTSD symptomology. Even though conclusions regarding the direction of this association should not be made due to the cross-sectional character of the present study, it accords with previous theories proposing that the increased centrality of a traumatic event leads to more intrusive, more accessible and vivid memories, and thus a higher post-traumatic burden [22,23,29]. Additionally, memories that are central to one’s life story are more closely linked to the way people see themselves and others [23]. Findings on guilt are in accordance with some [26] but in contrast to other [23] previous research on other populations, suggesting that women who feel guilty for experiencing FGM/C are at a higher risk for developing PTSD. Since the emotional processing of FGM/C (guilt and centrality in life) plays an important role in the emergence of PTSD symptoms, it needs to be considered in the treatment of women affected by FGM/C. However, trauma-processing interventions must be further evaluated in the FGM/C context [40,41,42,43].

Some limitations should be taken into consideration when interpreting the results of the present study: only women who sought medical advice were examined, limiting the generalizability of the present study. Additionally, even though the women were instructed to refer to FGM/C when asked for PTSD symptoms, other stressful events (e.g., sexual violence, forced marriage, war or flight) that were not assessed as part of this study could also play a role in the emergence of PTSD. Further research should include more comprehensive personal medical histories, and take into consideration a possible re-activation of trauma. Furthermore, the circumstances under which FGM/C took place (e.g., loss of trust in the most important caregivers and family members; medical complications) and other important characteristics (e.g., control beliefs or social support) were not assessed in the present study. In addition, the psychological symptoms were accessed with the help of screening instruments. This allows a suspicion to be expressed but does not permit a diagnosis to be established. Moreover, the questionnaires dispensed were in English, and while the help of a Somali language mediator was used when necessary, even though she had many years of experience in translating during FGM/C-related clinical investigations, she was not a trained interpreter. Additionally, women who did not speak Somali, English or German brought their own language mediator, limiting the standardization of the translations. Accordingly, it cannot be ruled out that items were left blank or incorrectly answered due to intensive feelings (e.g., shame). It would have been advantageous to use questionnaires in the women’s mother tongue (e.g., [44]). However, the existing questionnaires specially developed for an FGM/C context are still not sufficiently validated. Moreover, mediating factors such as cultural beliefs and the differentiation between women living in their home country and women living within western culture need to be further examined, based on a larger sample size. Given a larger sample size and a longitudinal study design, further research should conduct a structural equations model (SEM) to better understand latent factors and the process by which model variables are related, as well as how various factors moderate the strength and direction of the model variables as they affect one another.

## 5. Conclusions

The present study expands the limited knowledge about the emergence of PTSD in women suffering from FGM/C. These women represent a population posing considerable challenges for inclusion in structured research schemes. Physicians should be sensitized and trained for the special context of FGM/C to identify aspects of the women’s cognitive–emotional processing. This requires the development of screening instruments for patient selection in various languages, and well-evaluated interventions, not only in a medical but also in a psychosocial context. These interventions should be based on well-established treatments for PTSD, and extended by further research on the complex socio-cultural aspects of FGM/C. Future research should also investigate other predictors, such as the process of mutilation, medical complications and the social support and acceptance of FGM/C, which could also play a role in trauma processing. Longitudinal studies should be used to back up the findings of this study.

## Figures and Tables

**Table 1:** Excerpt of socio-demographic data of participants.

	Total (*N* = 112)		Suspected PTSD (*n* = 62)		No Suspected PTSD (*n* = 50)		Test Statistic *χ*^2^ (df); *t* (df); *n*	*p*	*d*/Cramer’s *V*
	*n*	%	*n*	%	*n*	%			
Age in Years							*t*_(110)_ = 0.03 *n* = 112	0.974	*d* = 0.01
*M*	28.11		28.08		28.14				
*SD*	9.37		8.78		10.14				
Range	14–63		14–57		15–63				
Children							*χ*^2^*_(_*_1)_ = 0.21 *n* = 112	0.705	*V* = 0.04
Yes	52	46.4	30	48.4	22	44.0			
No	60	53.6	32	51.6	28	56.0			
Marital status							*χ*^2^ = 3.26 ^b^ *n* = 110	0.528	*V* = 0.17
Single	56	50.0	28	45.2	28	56.0			
Married/domestic relationship	26	23.2	16	25.8	10	20.0			
Widowed	6	5.6	4	6.5	2	4.0			
Divorced or separated	22	19.6	14	22.6	8	16.0			
Missing	2	1.8	0	0.0	2	4.0			
Country of origin							*χ*^2^ = 25.29 ^b^ *n* = 110	0.009	*V* = 0.50
Somalia	72	64.3	42	67.7	30	60.0			
Nigeria	7	6.3	1	1.6	6	12.0			
Guinea	5	4.5	5	8.1	0	0.0			
Ethiopia	3	2.7	0	0.0	3	6.0			
Kenya	3	2.7	2	3.2	1	2.0			
Other countries ^a^	20	17.9	11	17.7	9	18.0			
Missing	2	1.8	1	1.6	1	2.0			
Religion							*χ*^2^ = 2.93 ^b^ *n* = 112	0.210	*V* = 0.16
Muslim	96	85.7	56	90.3	40	80.0			
Christian	15	13.4	6	9.7	9	18.0			
other	1	0.9	0	0.0	1	2.0			
Months in Germany							*t*_(59.2)_ = 2.43 ^c^ *n* = 102	0.018	*d* = −0.40
*M*	38.81		27.59		53.60				
*SD*	50.67		32.49		65.11				
Range	1–316		2–212		1–316				
Missing	10	8.9	4	3.6	6	12.0			
Employment status							*χ*^2^ = 7.03 ^b^ *n* = 111	0.067	*V* = 0.25
Unemployed	63	56.3	41	66.1	22	44.0			
Employed full-time	8	7.1	2	3.2	6	12.0			
Employed part-time	9	8.0	4	6.5	5	10.0			
Student	31	27.7	14	22.6	17	34.0			
Missing	1	0.9	1	1.6	0	0.0			
Knowledge about female anatomy (medical opinion)							*χ*^2^ = 5.24 ^b^ *n* = 111	0.140	*V* = 0.22
No knowledge	85	75.9	50	80.6	35	70.0			
Low	17	15.2	9	14.5	8	16.0			
Moderate	7	6.3	1	1.6	6	12.0			
High	2	1.8	1	1.6	1	2.0			
Missing	1	0.9	1	1.6	0	0.0			
Type of FGM (medical opinion)							*χ*^2^*_(_*_2)_ = 0.63 *n* = 112	0.721	*V* = 0.08
Type I	21	18.8	10	16.1	11	22.0			
Type II	35	31.3	20	32.3	15	30.0			
Type III	56	50.0	32	51.6	24	48.0			
Age at FGM							*t*_(110)_ = −2.87 *n* = 112	0.005	*d* = 0.51
*M*	7.33		8.14		6.17				
*SD*	3.74		3.39		3.90				
Range	0–21		0–21		0–12				
Missing	11	9.8	0	0.0	0	0.0			
Suspected depression (PHQ-2 ≥ 3)							*χ*^2^_(1)_ = 4.38 *n* = 112	0.049	*V* = 0.20
Yes	41	36.6	28	45.2	13	26.0			
No	71	63.4	34	54.8	37	74.0			
Suspected anxiety disorder (GAD-2 ≥ 3)							*χ*^2^_(1)_ = 14.78 *n* = 112	0.000	*V* = 0.36
Yes	54	48.2	40	64.5	14	28.0			
No	58	51.8	22	35.5	36	72.0			
Suspected PTSD (PC-PTSD-5 ≥ 3)									
Yes	62	55.4							
No	50	44.6							
Suspected depression (PHQ-2 ≥ 3) and suspected anxiety disorder (GAD-2 ≥ 3)									
Yes	35	31.3	26	41.9	9	18.0	*χ*^2^_(1)_ = 7.38 *n* = 112	0.008	*V* = 0.26
No	77	68.8	36	58.1	41	82.0			

Notes. Total = all participants; Suspected PTSD = participants with suspected PTSD (PC-PTSD-5 ≥ 3); No suspected PTSD = participants without suspicion of PTSD (PC-PTSD-5 < 3); ^a^ Egypt (*n* = 2; 1.8%), Burkina Faso (*n* = 2; 1.8%), Ivory Coast (*n* = 2; 1.8%), Eritrea (*n* = 2; 1.8%), Gambia (*n* = 2; 1.8%), Sudan (*n* = 2; 1.8%), Liberia (*n* = 1; 0.9%), Qatar (*n* = 1; 0.9%), Senegal (*n* = 1; 0.9%), Sierra Leone (*n* = 1; 0.9%), Great Britain (*n* = 1; 0.9%), Chad (*n* = 1; 0.9%); Djibouti (*n* = 1; 0.9%); Iraq (*n* = 1; 0.9%); ^b^ Fischer’s exact test; ^c^ Welch test; after Bonferroni correction, values *p* < 0.003 are considered significant. More socio-demographic characteristics of the sample can be found in Table S1.

**Table 2:** Excerpt of physical health.

	Total (*N* = 112)		Type I *(n* = 21)		Type II (*n* = 35)		Type III (*n* = 56)		Test Statistic *χ*^2^ (df); *t* (df); *n*	*p*	*d*/Cramer’s *V*
	*n*	%	*n*	%	*n*	%	*n*	%			
Ever having pain in abdomen (womb or ovaries, apart from period pains)									*χ*^2^ = 8.70 ^c^ *n* = 112	0.185	0.21
Never	23	20.5	2	9.5	12	34.3	9	16.1			
Rarely	22	19.6	7	33.3	5	14.3	10	17.9			
Often	47	42	10	47.6	12	34.3	25	44.6			
Always	20	17.9	2	9.5	6	17.1	12	21.4			
Ever having gynecological infections (vagina, womb or ovaries)									*χ*^2^ = 6.47 ^c^ *n* = 109	0.36	0.18
Never	69	61.6	11	52.4	23	65.7	35	62.5			
Rarely	19	17	4	19	7	20	8	14.3			
Often	2	10.7	2	9.5	2	5.7	8	14.3			
Always	9	8	4	19	3	8.6	2	3.6			
Missing	3	2.7	0	0	0	0	3	5.4			
Ever having sexually transmittable disease									*χ*^2^ = 10.36 ^c^ *n* = 111	0.015	0.28
Never	104	92.9	17	81	35	100	52	92.9			
Rarely	3	2.7	2	9.5	0	0	1	1.8			
Often	2	1.8	0	0	0	0	2	3.6			
Always	2	1.8	2	9.5	0	0	0	0			
Missing	1	0.9	0	0	0	0	1	1.8			
Ever having period pain									*χ*^2^ = 5.55 ^c^ *n* = 112	0.473	0.16
Never	41	36.6	6	28.6	13	37.1	22	39.3			
Rarely	8	7.1	3	14.3	3	8.6	2	3.6			
Often	27	24.1	7	33.3	9	25.7	11	19.6			
Always	36	32.1	5	23.8	10	28.6	21	37.5			
Ever having stagnation of blood in vagina or womb during period									*χ*^2^ = 7.34 ^c^ *n* = 109	0.272	0.19
Never	73	65.2	11	52.4	26	74.3	36	64.3			
Rarely	16	14.3	5	23.8	3	8.6	8	14.3			
Often	11	9.8	1	4.8	4	11.4	6	10.7			
Always	9	8	4	19	1	2.9	4	7.1			
Missing	3	2.7	0	0	1	2.9	2	3.6			
Ever having pain urinating									*χ*^2^ = 3.47 ^c^ *n* = 109	0.765	0.13
Never	58	51.8	9	42.9	19	54.3	30	53.6			
Rarely	26	23.2	7	33.3	8	22.9	11	19.6			
Often	14	12.5	1	4.8	5	14.3	8	14.3			
Always	11	9.8	3	14.3	3	8.6	5	8.9			
Missing	3	2.7	1	4.8	0	0	2	3.6			
Ever having urinary infections									*χ*^2^ = 3.89 ^c^ *n* = 109	0.708	0.12
Never	72	64.3	11	52.4	23	65.7	38	67.9			
Rarely	19	17	5	23.8	6	17.1	8	14.3			
Often	11	9.8	3	14.3	4	11.4	4	7.1			
Always	7	6.3	2	9.5	1	2.9	4	7.1			
Missing	3	2.7	0	0	1	2.9	2	3.6			
Ever strained to empty bladder									*χ*^2^ = 10.72 ^c^ *n* = 108	0.078	0.23
Never	69	61.6	12	57.1	26	74.3	31	55.4			
Rarely	11	9.8	1	4.8	4	11.4	6	10.7			
Often	10	8.9	1	4.8	0	0	9	16.1			
Always	18	16.1	6	28.6	5	14.3	7	12.5			
Missing	4	3.6	1	4.8	0	0	3	5.4			
Body function that has caused the severest health complaints or impairments									*χ*^2^ = 6.20 ^c^ *n* = 108	0.175	0.18
Gynecology	25	22.3	6	28.6	7	20	12	21.4			
Menstruation	70	62.5	14	66.7	18	51.4	38	67.9			
Bladder	13	11.6	1	4.8	8	22.9	4	7.1			
Missing	4	3.6	0	0	2	5.7	2	3.6			
Impairment in daily life									*χ*^2^ = 12.63 ^c^ *n* = 112	0.044	0.24
Not at all	26	23.2	6	28.6	12	34.3	8	14.3			
A little	18	16.1	5	23.8	4	11.4	9	16.1			
Quite a lot	34	30.4	4	19	14	40	16	28.6			
Very much	34	30.4	6	28.6	5	14.3	23	41.1			
Level of stress during health complaints ^a^									*F*_(2)_ = 0.53	0.59	
*M*	6.6		6.05		6.91		6.61		*n* = 112		
*SD*	3.04		3.41		2.83		3.04				
Range	0–10		0–10		0–10		0–10				
Level of pain during health complaint ^b^									*F_(_*_2)_ = 1.02	0.366	
*M*	6.89		6.95		6.37		7.2		*n* = 112		
*SD*	2.7		3.09		3.07		2.26				
Range	0–10		0–10		0–10		1–10				
Withdrawing from other people									*χ*^2^ = 4.11 ^c^ *n* = 110	0.673	0.14
Not at all	37	33	8	38.1	15	42.9	14	25			
A little	30	26.8	6	28.6	9	25.7	15	26.8			
Quite a lot	23	20	4	19	7	20	12	21.4			
Very much	20	17.9	3	14.3	4	11.4	13	23.2			
Missing	2	1.8	0	0	0	0	2	3.6			

Notes. Total = all participants; Type I = participants with type I; Type II = participants with type II; Type III = participants with type III; ^a^ from 0 = no stress to 10 = worst stress imaginable; ^b^ from 0 no pain to 10 = worst pain imaginable; ^c^ Fischer’s exact test; after Bonferroni correction, values *p* < 0.004 are considered significant. Additional medical complications of the sample can be found in Table S2.

**Table 3:** Predictors for PTSD.

	B	95% CI	SE B	Β	*t*	*p*
**Step 1**						
Constant	1.71	0.69; 2.72	0.51		3.34	0.001
Dummy Type II	0.21	−0.77; 1.18	0.49	0.05	0.42	0.673
Dummy Type III	0.09	−0.82; 0.99	0.46	0.03	0.20	0.845
Age at FGM	0.11	0.02; 0.20	0.05	0.23	2.45	0.016
**Step 2**						
Constant	−1.10	−2.41; 0.21	0.66		−1.67	0.100
Dummy Type II	0.61	-0.21; 1.44	0.42	0.16	1.47	0.145
Dummy Type III	0.23	−0.53; 1.00	0.38	0.06	0.60	0.552
Age at FGM	0.09	0.02; 0.17	0.04	0.19	2.43	0.017
Shame	0.07	−0.00; 0.13	0.03	0.20	2.00	0.052
Guilt	0.10	0.03; 0.16	0.03	0.32	3.05	0.003
CES	0.05	0.01; 0.08	0.02	0.19	2.32	0.022
**Step 3**						
Constant	0.28	−2.00; 2.54	1.214		0.25	0.804
Dummy Type II	0.52	−0.31; 1.35	0.42	0.13	1.24	0.218
Dummy Type III	0.20	−0.56; 0.95	0.38	0.05	0.52	0.608
Age at FGM	0.08	0.00; 0.16	0.04	0.16	2.03	0.045
Shame	0.05	−0.02; 0.12	0.04	0.15	1.38	0.172
Guilt	0.09	0.03; 0.16	0.03	0.30	2.83	0.006
CES	0.04	0.00; 0.08	0.02	0.17	2.14	0.035
Body Image	−0.03	−0.07; 0.01	0.02	−0.14	−1.48	0.142

Notes. Step 1: R^2^ = 0.06, adjusted R^2^ = 0.03 (*p* = 0.105); Step 2: R^2^ = 0.36, adjusted R^2^ = 0.32, ∆R^2^ = 0.30, (*p* < 0.001); Step 3: R^2^ = 0.37, adjusted R^2^ = 0.33, ∆R^2^ = 0.01, (*p* = 0.142); CES = Centrality of Event Scale.

## Data Availability

Individual anonymized participant data will not be shared due to the privacy of the participating women. Statistical analyses are available on request from the corresponding author.

## Supplementary Materials

The following supporting information can be downloaded at: https://www.mdpi.com/article/10.3390/ijerph19094993/s1, Table S1: Socio-demographic data of participants; Table S2: Physical health; Table S3: Pearson’s correlation coefficients.
