# Supplementary material for: Cognitive–Emotional Aspects of Post-Traumatic Stress Disorder in the Context of Female Genital Mutilation
Source: Int J Environ Res Public Health. 2022 Apr 20;19(9):4993. doi: 10.3390/ijerph19094993 (PMC9105982; doi:10.3390/ijerph19094993)
Supplement: Supplementary file 1 [file ijerph-19-04993-s001.zip › ijerph-1638684-supplementary.pdf]

Supplemental Material

Table S1. Socio-Demographic Data of Participants.

	total (*N* = 112)		Suspected PTSD (*n* = 62)		No suspected PTSD (*n* = 50)		Test statistic χ^2 (df); t (df); *n*	*p*	*d*/ Cramer's *V*
	n	%	*n*	%	*n*	%			
Age in Years							$t_{(110)}$ =	0.974	d =
M	28.11		28.08		28.14		0.03		0.01
SD	9.37		8.78		10.14		*n* = 112		
Range	14–63		14–57		15–63				
Children								0.705	V = 0.04
Yes	52	46.4	30	48.4	22	44.0	$\chi^2_{(1)}$ = 0.21		
No	60	53.6	32	51.6	28	56.0	*n* = 112		
Number of children							$t_{(106)}$ =	0.372	d =
M	1.38		1.52		1.19		−0.90		0.20
SD	1.91		2.12		1.61		*n* = 108		
Range	0–8		0–8		0–6				
Missing	4	3.6	1	1.6	3	6			
Marital status								0.528	V = 0.17
Single	56	50.0	28	45.2	28	56.0	χ^2 = 3.26[b]		
Married/ domestic relationship	26	23.2	16	25.8	10	20.0	*n* = 110		
Widowed	6	5.6	4	6.5	2	4.0			
Divorced or separated	22	19.6	14	22.6	8	16.0			
Missing	2	1.8	0	0.0	2	4.0			
Highest level of education							χ^2 = 3.76[b]	0.616	V = 0.19
No school	50	44.6	30	48.4	20	40.0	*n* = 112		
Less than high school degree	33	29.5	19	30.6	14	280			
High school degree or equivalent	6	5.4	3	4.8	3	6.0			
apprenticeship or some college, but no degree	7	6.3	3	4.8	4	8.0			
College degree	16	14.3	7	11.3	9	18.0			
Country of origin							χ^2 = 25.29[b]	0.009	V = 0.50
Somalia	72	64.3	42	67.7	30	60.0	*n* = 110		
Nigeria	7	6.3	1	1.6	6	12.0			
Guinea	5	4.5	5	8.1	0	0.0			
Ethiopia	3	2.7	0	0.0	3	6.0			
Kenya	3	2.7	2	3.2	1	2.0			
Egypt	2	1.8	1	1.6	1	2.0			
Burkina Faso	2	1.8	2	3.2	0	0.0			
Ivory Coast	2	1.8	1	1.6	1	2.0			
Eritrea	2	1.8	0	0.0	2	4.0			
Gambia	2	1.8	1	1.6	1	2.0			
Sudan	2	1.8	2	3.2	0	0.0			
Other countries [a]	8	7.1	4	6.5	4	8.0			
Missing	2	1.8	1	1.6	1	2.0			
Type of region in home country							χ^2 = 5.96[b]	0.052	V = 0.24
urban	45	40.2	19	30.6	26	52.0	*n* = 106		
rural	54	48.2	36	58.1	18	36.0			
suburban	7	6.3	4	6.5	3	6.0			

Missing	6	5.4	3	4.8	3	6.0			
religion								0.210	V = 0.16
Muslim	96	85.7	56	90.3	40	80.0	χ^2 = 2.93[b]		
Christian	15	13.4	6	9.7	9	18.0	*n* = 112		
other	1	0.9	0	0.0	1	2.0			
Current country							χ^2 = [b]	0.464	V = 0.01
Germany	103	920	58	93.5	45	90.0	*n* = 111		
others	8	7.1	3	4.8	5	10.0			
Missing	1	0.9	1	1.6	0	0.0			
Months in Germany								0.018	d =
M	38.81		27.59		53.60		$t_{(59.2)}$ = 2.43[c]		−0.40
SD	50.67		32.49		65.11		*n* = 102		
Range	1–316		2–212		1–316				
Missing	10	8.9	4	3.6	6	12.0			
Status of asylum procedure							χ^2 = 10.94[b]	0.047	V = 0.34
Permanent right of residence	10	8.9	3	4.8	7	14.0	*n* = 99		
Temporary resident permit	29	25.9	14	22.6	15	30.0			
No decisions yet concerning asylum application	23	20.5	15	24.2	8	16.0			
Registered but no asylum application yet	20	17.9	15	24.2	5	10.0			
No asylum application	6	5.4	2	3.2	4	8.0			
Asylum application denied, obligation to depart	11	9.8	9	14.5	2	4.0			
Missing	13	11.6	4	6.5	9	18.0			
Employment status								0.067	V = 0.25
Unemployed	63	56.3	41	66.1	22	44.0	χ^2 = 7.03[b]		
Employed full time	8	7.1	2	3.2	6	12.0	*n* = 111		
Employed part time	9	8.0	4	6.5	5	10.0			
Student	31	27.7	14	22.6	17	34.0			
Missing	1	0.9	1	1.6	0	0.0			
Knowledge about female anatomy (medical opinion)								0.140	
No knowledge	85	75.9	50	80.6	35	70.0	χ^2 = 5.24[b]		V = 0.22
Low	17	15.2	9	14.5	8	16.0	*n* = 111		
Moderate	7	6.3	1	1.6	6	12.0			
High	2	1.8	1	1.6	1	2.0			
Missing	1	0.9	1	1.6	0	0.0			
Type of FGM (medical opinion)								0.721	V = 0.08
Type I	21	18.8	10	16.1	11	22.0	$\chi^2_{(2)}$ = 0.63		
Type II	35	31.3	20	32.3	15	30.0	*n* = 112		
Type III	56	50.0	32	51.6	24	48.0			
Age at FGM							$t_{(110)}$ =	0.005	d =
M	7.33		8.14		6.17		-2.87		.51
SD	3.74		3.39		3.90		*n* = 112		
Range	0–21		0–21		0–12				
Missing	11	9.8	0	0.0	0	0.0			
FGM demanded by religion (subjective)								0.811	V = 0.05
Yes	22	19.6	11	17.7	11	22.0	$\chi^2_{(1)}$ = 0.23		
No	88	78.6	49	79.0	39	78.0	*n* = 110		
Missing	2	1.8	2	3.2	0	0.0			
Considering reconstructive surgery							$\chi^2_{(1)}$ = 0.66	0.440	V = 0.08

Yes	58	51.8	31	50.0	27	54.0	$n = 107$		
No	49	43.8	30	48.4	19	38.0			
Missing	5	4.5	1	1.6	4	8.0			
Suspected depression (PHQ-2 ≥ 3)								0.049	
Yes	41	36.6	28	45.2	13	26.0	$\chi^2_{(1)} = 4.38$		V = 0.20
No	71	63.4	34	54.8	37	74.0	$n = 112$		
Suspected anxiety disorder (GAD-2 ≥ 3)								0.000	
Yes	54	48.2	40	64.5	14	28.0	$\chi^2_{(1)} = 14.78$		V = 0.36
No	58	51.8	22	35.5	36	72.0	$n = 112$		
Suspected PTSD (PC-PTSD-5 ≥ 3)									
Yes	62	55.4							
No	50	44.6							
Suspected depression (PHQ-2 ≥ 3) and suspected anxiety disorder (GAD-2 ≥ 3)									
Yes	35	31.3	26	41.9	9	18.0	$\chi^2_{(1)} = 7.38$	0.008	V = 0.26
No	77	68.8	36	58.1	41	82.0	$n = 112$		

Note. Total = all participants; Suspected PTSD = participants with suspected PTSD (PC-PTSD-5 ≥ 3); No suspected PTSD = participants without suspicion of PTSD (PC-PTSD-5 <3); [a] Liberia (*n* = 1; 0.9%), Qatar (*n* = 1; 0.9%), Senegal (*n* = 1; 0.9%), Sierra Leone (*n* = 1; 0.9%), Great Britain (*n* = 1; 0.9%), Chad (*n* = 1; 0.9%); Djibouti (*n* = 1; 0.9%); Iraq (*n* = 1; 0.9%); [b] Fischer's exact test; [c] Welch test; after Bonferroni correction, values $p < 0.003$ are considered significant.

Table S2. Physical Health.

	total (*N* = 112)		Type I (*n* =21)		Type II (*n* = 35)		Type III (*n* = 56)		Test statistic χ^2 (df); t (df); *n*	*p*	*d*/ Cramer's *V*
	n	**%**	***n***	**%**	***n***	**%**	***n***	**%**			
Ever having pain in abdomen (womb or ovaries, apart from period pains)									$\chi^2 = 8.70^c$ *n* = 112	0.185	0.21
Never	23	20.5	2	9.5	12	34.3	9	16.1			
Rarely	22	19.6	7	33.3	5	14.3	10	17.9			
Often	47	42.0	10	47.6	12	34.3	25	44.6			
Always	20	17.9	2	9.5	6	17.1	12	21.4			
Ever having gynecological infections (vagina, womb or ovaries)										0.360	0.18
Never	69	61.6	11	52.4	23	65.7	35	62.5	$\chi^2 = 6.47^c$ *n* = 109		
Rarely	19	17.0	4	19.0	7	20.0	8	14.3			
Often	2	10.7	2	9.5	2	5.7	8	14.3			
Always	9	8.0	4	19.0	3	8.6	2	3.6			
Missing	3	2.7	0	0.0	0	0.0	3	5.4			
Ever having sexually transmittable disease										0.015	0.28
Never	104	92.9	17	81.0	35	100.0	52	92.9	$\chi^2 = 10.36^c$ *n* = 111		
Rarely	3	2.7	2	9.5	0	0.0	1	1.8			
Often	2	1.8	0	0.0	0	0.0	2	3.6			
Always	2	1.8	2	9.5	0	0.0	0	0.0			
Missing	1	0.9	0	0.0	0	0.0	1	1.8			
Ever having period pain										0.473	0.16
Never	41	36.6	6	28.6	13	37.1	22	39.3	$\chi^2 = 5.55^c$ *n* = 112		
Rarely	8	7.1	3	14.3	3	8.6	2	3.6			
Often	27	24.1	7	33.3	9	25.7	11	19.6			
Always	36	32.1	5	23.8	10	28.6	21	37.5			
Ever having stagnations of blood in vagina or womb during period										0.272	0.19
Never	73	65.2	11	52.4	26	74.3	36	64.3	$\chi^2 = 7.34^c$ *n* = 109		
Rarely	16	14.3	5	23.8	3	8.6	8	14.3			
Often	11	9.8	1	4.8	4	11.4	6	10.7			
Always	9	8.0	4	19.0	1	2.9	4	7.1			
Missing	3	2.7	0	0.0	1	2.9	2	3.6			
Ever having pain urinating										0.765	0.13
Never	58	51.8	9	42.9	19	54.3	30	53.6	$\chi^2 = 3.47^c$ *n* = 109		
Rarely	26	23.2	7	33.3	8	22.9	11	19.6			
Often	14	12.5	1	4.8	5	14.3	8	14.3			
Always	11	9.8	3	14.3	3	8.6	5	8.9			
Missing	3	2.7	1	4.8	0	0.0	2	3.6			
Ever having urinary infections										0.708	0.12
Never	72	64.3	11	52.4	23	65.7	38	67.9	$\chi^2 = 3.89^c$ *n* = 109		
Rarely	19	17.0	5	23.8	6	17.1	8	14.3			
Often	11	9.8	3	14.3	4	11.4	4	7.1			
Always	7	6.3	2	9.5	1	2.9	4	7.1			
Missing	3	2.7	0	0.0	1	2.9	2	3.6			
Ever strained to empty bladder										0.078	
Never	69	61.6	12	57.1	26	74.3	31	55.4			
Rarely	11	9.8	1	4.8	4	11.4	6	10.7	$\chi^2 = 10.72^c$ *n* = 108		0.23
Often	10	8.9	1	4.8	0	0.0	9	16.1			
Always	18	16.1	6	28.6	5	14.3	7	12.5			
Missing	4	3.6	1	4.8	0	0.0	3	5.4			

Body function that has caused the severest health complaints or impairments									$\chi^2 = 6.20^c$ $n = 108$	0.175	0.18
Gynecology	25	22.3	6	28.6	7	20.0	12	21.4			
Men-struation	70	62.5	14	66.7	18	51.4	38	67.9			
Bladder	13	11.6	1	4.8	8	22.9	4	7.1			
Missing	4	3.6	0	0.0	2	5.7	2	3.6			
Impairment in daily life										0.044	0.24
Not at all	26	23.2	6	28.6	12	34.3	8	14.3	$\chi^2 = 12.63^c$		
A little	18	16.1	5	23.8	4	11.4	9	16.1	$n = 112$		
Quite a lot	34	30.4	4	19.0	14	40.0	16	28.6			
Very much	34	30.4	6	28.6	5	14.3	23	41.1			
Withdrawing from other people										0.673	0.14
Not at all	37	33.0	8	38.1	15	42.9	14	25.0	$\chi^2 = 4.11^c$		
A little	30	26.8	6	28.6	9	25.7	15	26.8	$n = 110$		
Quite a lot	23	20.	4	19.0	7	20.0	12	21.4			
Very much	20	17.9	3	14.3	4	11.4	13	23.2			
Missing	2	1.8	0	0.0	0	0.0	2	3.6			
Knowledge of female anatomy (medical opinion)									$\chi^2 = 6.51^c$ $n = 111$	0.308	0.18
No knowledge	85	75.9	18	85.7	22	62.9	45	80.4			
Low	17	15.2	3	14.3	6	17.1	8	14.3			
Moderate	7	6.3	0	0.0	5	14.3	2	3.6			
High	2	1.8	0	0.0	1	2.9	1	1.8			
Missing	1	0.9	0	0.0	1	2.9	0	0.0			
Other peculiarities in her genital area (medical opinion; multiple answers possible)											
None	9	8.0	1	4.8	3	8.6	5	8.9			
Neuroma/ granuloma	6	5.4	1	4.8	1	2.9	4	7.1			
Scars	90	80.4	18	85.7	27	77.1	45	80.4			
Others	1	0.9	1	4.8	0	0.0	0	0.0			
Missing	11	9.8	1	4.8	5	14.3	5	8.9			
Level of stress during health complaints [a]									$F_{(2)} =$ 0.53 $n = 112$	0.590	
M		6.60		6.05		6.91		6.61			
SD		3.04		3.41		2.83		3.04			
Range		0–10		0–10		0–10		0–10			
Level of pain during health complaints [b]									$F_{(2)} =$ 1.02 $n = 112$	0.366	
M		6.89		6.95		6.37		7.20			
SD		2.70		3.09		3.07		2.26			
Range		0–10		0–10		0–10		0–10			

Notes. Total = all participants; Type I = participants with type I; Type II = participants with type II; Type III = participants with type III; [a] from 0 = no stress to 10 = worst stress imaginable; [b] from 0 no pain to 10 = worst pain imaginable; [c] Fischer's exact test; after Bonferroni correction, values $p < 0.004$ are considered significant.

Table S3. Pearson's correlation coefficients (*N* = 112).

	Predictor	*M*	*SD*	1	2	3	4	5	6	7
1.	PC-PTSD-5	2.62	1.81	-						
2.	Type II	0.31	0.47	0.05	-					
3.	Type III	0.50	0.50	−0.02	−0.67***	-				
4.	Age at FGM	7.26	3.74	0.23**	0.05	−0.05	-			
5.	SSGS Shame	10.58	5.54	0.45***	−0.11	−0.03	0.09	-		
6.	SSGS Guilt	9.38	5.98	0.49***	−0.14	0.05	0.03	0.65***	-	
7.	CES	24.95	7.53	0.31***	−0.06	0.04	0.05	0.16*	0.26**	-
8.	Body image	31.31	8.82	−0.42***	−0.07	0.04	−0.24**	−0.50***	−0.42***	−0.21*

Note. * $p < 0.05$; ** $p < 0.01$; *** $p < 0.001$; PC-TPSD-5 = Primary Care PTSD Screen for DSM-5; SSGS = State Shame and Guilt Scale; CES = Centrality of Event Scale.
